# Marker tolerant, immunocompetent animals as a new tool for regenerative medicine and long-term cell tracking

**DOI:** 10.1186/1472-6750-7-30

**Published:** 2007-06-08

**Authors:** Kathrin I Odörfer, Nina J Unger, Karin Weber, Eric P Sandgren, Reinhold G Erben

**Affiliations:** 1 Institute of Pathophysiology, Dept. of Natural Sciences, University of Veterinary Medicine, Vienna 1210, Austria; 2 Institute of Physiology, Physiological Chemistry and Animal Nutrition, Ludwig Maximilians University, Munich 80539, Germany; 3 Department of Pathobiological Sciences, School of Veterinary Medicine, University of Wisconsin-Madison, Madison, Wisconsin 53706, USA

## Abstract

**Background:**

Immune-mediated rejection of labeled cells is a general problem in transplantation studies using cells labeled with any immunogenic marker, and also in gene therapy protocols. The aim of this study was to establish a syngeneic model for long-term histological cell tracking in the absence of immune-mediated rejection of labeled cells in immunocompetent animals. We used inbred transgenic Fischer 344 rats expressing human placental alkaline phosphatase (hPLAP) under the control of the ubiquitous R26 promoter for this study. hPLAP is an excellent marker enzyme, providing superb histological detection quality in paraffin and plastic sections.

**Results:**

Transplantation of cells from hPLAP transgenic (hPLAP-tg) F344 rats into wild-type (WT) F344 recipients failed because of immune-mediated rejection. Here we show that this problem can be overcome by inducing tolerance to the marker gene by transplantation of bone marrow from hPLAP-tg F344 rats into WT F344 hosts after lethal irradiation, or by neonatal exposure of WT F344 rats to hPLAP-tg F344 cells. As proof-of-principle, we injected bone marrow cells (BMC) from hPLAP-tg rats into the knee joint of marker tolerant, bone marrow-transplanted WT rats, and found successful engraftment and differentiation of donor cells. In addition, hPLAP-tg BMC injected intravenously in neonatally tolerized WT F344 hosts could be traced in lymph nodes, 2 months post-injection.

**Conclusion:**

In combination with the excellent marker hPLAP, marker tolerant animals may open up new perspectives for all experiments requiring long-term histological tracking of genetically labeled cells.

## Background

Cell therapy or cell-based gene therapy with adult pluripotent mesenchymal stem cells is thought to revolutionize the treatment of a large variety of diseases of various organ systems in the future [reviewed in [1]]. To further explore the therapeutic potential of regenerative treatment protocols, appropriate animal models are necessary that allow tracing the fate of individual donor or manipulated cells in the host organism.

Tracing of cells requires labeling, and one standard approach to label cells is to introduce marker genes into the genome of the cells under investigation. Marker genes can either be permanently integrated into the genome of transgenic animals so that all or at least some somatic cells are permanently labeled, or wild-type cells can be transduced with vectors containing the marker gene. A stable genetic marker is especially useful for cell lineage experiments, because the marker is expressed in whole progeny of a specific cell.

Recent work from our laboratory has shown that human placental alkaline phosphatase (hPLAP) is a highly suitable marker enzyme for studies involving genetically labeled cells in all tissues, including hard tissues, because it survives not only paraffin but also modified methylmethacrylate (MMA) embedding [2,3]. hPLAP is a heat-stable enzyme that is developmentally neutral in transgenic rats and mice [4]. In addition, endogenous alkaline phosphatase activity can be totally blocked by heat inactivation. Thus, this marker enzyme provides superb detection quality of labeled cells in the total absence of background staining.

The present experiments employ R26-hPLAP transgenic inbred Fischer 344 (hPLAP-tg) rats. The R26 promoter represents a 0.8 kb fragment of the ROSA βgeo 26 (ROSA26) promoter sequence which has been found to be especially useful to direct ubiquitous expression of marker genes in mice and rats [4,5]. Transgenic mice and rats expressing hPLAP under the control of the R26 promoter show ubiquitous, uniform, and stable expression of this genetic marker [4]. Therefore, hPLAP-tg F344 rats were expected to be a highly useful model for labeling donor cells in syngeneic transplantation studies [4].

However, during recent years, it has become increasingly clear that membrane or even intracellular expression of any foreign protein, and, thus, of any marker protein, will elicit immune-mediated rejection of transplanted cells carrying the marker gene in the recipients [6-10]. Therefore, immune-mediated rejection of genetically altered cells is a general and very significant problem in transplantation studies using cells labeled with any marker gene, and also in gene therapy protocols. This problem has severely hampered the utility of animal models aimed at testing the usefulness of cell and gene therapy, especially in long-term studies. Here we describe a novel *in vivo *technology for studying labeled cells in the complete absence of immune-mediated rejection in immunocompetent hosts.

## Results

### Immune response of wild-type rats after syngeneic transplantation of cells from hPLAP-tg rats

**Figure 1:**
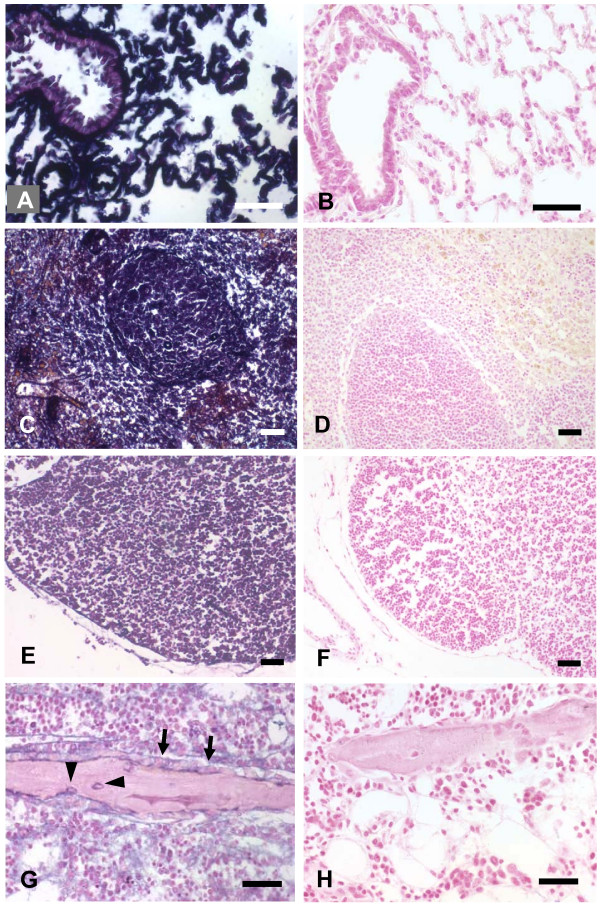
Transplantation of bone marrow cells (BMC) from hPLAP-tg rats into wild-type F344 recipients. Transgenic, hPLAP-labeled donor BMC were intravenously injected daily over 1 week into wild-type rats. Lung (A), spleen (C), and lymph nodes (E) from transgenic animals show strong and ubiquitous hPLAP staining. Similarly, osteocytes (arrowheads), osteoblasts (arrows), and all hematopoietic BMC show hPLAP staining in undecalcified MMA sections of ethanol-fixed tibias from hPLAP-tg rats (G). However, staining is completely absent in lung (B), spleen (D), lymph nodes (F), bone (H), and bone marrow (H) of wild-type rats intravenously injected with transgenic BMC over 1 week, 1 week after the last injection, indicating that syngeneic transplantation of transgenic BMC into wild-type recipients failed. The 5-μm-thick paraffin and MMA sections shown in A-H were stained for hPLAP enzyme activity overnight at room temperature after heat pretreatment, and were counterstained with nuclear fast red. Bar = 50 μm.

**Figure 2:**
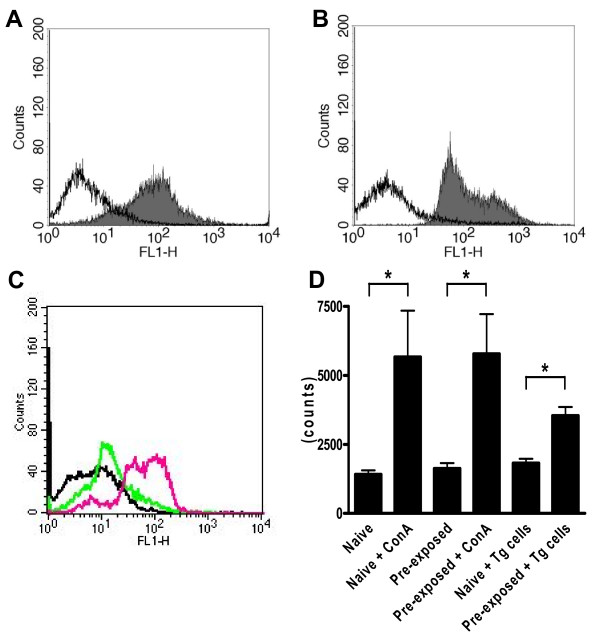
FACS analysis of peripheral blood and bone marrow cells from hPLAP-tg rats and immune response of wild-type rats to transgenic cells. The single parameter fluorescence histograms show hPLAP expression (mAb clone 8B6) on the majority of peripheral blood leukocytes (A) and practically all bone marrow cells (B) in hPLAP-tg rats (grey area), but no expression in wild-type rats (white area). When peripheral blood leukocytes from hPLAP-tg rats are incubated with serum from a naïve wild-type rat, and stained for bound antibodies, no staining is seen (black curve, C). However, serum from the same wild-type intraperitoneally injected with cells from transgenic donors 3 times shows strong reactivity with peripheral blood leukocytes from hPLAP-tg rats (C, green, after 2 injections; red, after 3 injections), indicating the induction of circulating antibodies against transgenic cells in the recipient. Ten-thousand cells were analyzed in each sample. In a mixed lymphocyte reaction protocol (D), co-culture of hPLAP-tg spleen cells with spleen cells from wild-type rats previously injected with cells from hPLAP-tg rats, but not from naïve wild-type rats, caused increased cell proliferation. ConA was used as a positive control. 5 × 10^5 ^spleen cells per well were cultivated for 5 days (n = 8 wells each). * denotes P < 0.05 vs. indicated control group by one-way ANOVA followed by least significant difference test.

In order to test whether peripheral blood cells and bone marrow cells from transgenic donors would survive and proliferate in normal wild-type rats of the same inbred strain, we intravenously injected peripheral blood leukocytes (PBL) or bone marrow cells (BMC) from hPLAP-tg donor rats daily for 1 week into wild-type F344 rats. However, histological analysis of lung, liver, spleen, lymph nodes, and bone failed to show any evidence of hPLAP-expressing cells from transgenic donors in the wild-type recipients, suggesting that it was not possible to syngeneically transplant cells from transgenic donors into wild-type rats (Fig. 1). This finding prompted us to ask the question whether expression of the transgene, and especially membrane expression of hPLAP, would induce the formation of antibodies to hPLAP in the wild-type recipients. FACS (Fluorescence-Activated Cell Sorter) analysis revealed that almost all PBL and BMC showed membrane expression of the hPLAP enzyme in hPLAP-tg rats (Fig. 2A and 2B). Immunization phenomena against hPLAP could result in immune-mediated destruction of any hPLAP-expressing cells in wild-type recipients, because the hPLAP enzyme represents a foreign protein to the rat immune system.

To test whether wild-type recipients develop antibodies against hPLAP after injection of cells from transgenic donor rats of the same strain, we intraperitoneally or subcutaneously injected wild-type F344 rats with PBL from hPLAP-tg rats three times with an 11-day interval between the injections. Subsequently, serum from naïve wild-type F344 rats or from wild-type F344 rats that had received injections of PBL from hPLAP-tg rats was incubated with PBL from transgenic rats, and the presence of antibody-labeled cells was examined by FACS analysis. As expected, serum from naïve wild-type rats that had never been in contact with transgenic cells did not react with PBL from transgenic rats (Fig. 2C). However, as shown in Fig. 2C, repeated intraperitoneal injection of transgenic PBL induced an antibody response in wild-type rats. Identical results were obtained with subcutaneous injection (data not shown). To test a cellular immune response to hPLAP in wild-type rats, we performed mixed lymphocyte reaction protocols. When spleen cells from naïve wild-type rats were co-cultured with transgenic spleen cells, we did not observe increased cell proliferation (Fig. 2D). However, cell proliferation was induced when spleen cells from wild-type rats that had previously been exposed to hPLAP-expressing cells were co-cultured with transgenic cells. These data demonstrate that exposure to hPLAP-tg cells induces immunization phenomena in wild-type rats, explaining the abovementioned failure of the experiments aimed at the syngeneic transplantation of transgenic cells into normal wild-type recipients. To verify this hypothesis, we transplanted skin from hPLAP-tg F344 rats into naïve, 3-month-old wild-type F344 recipients (n = 3). All recipients rejected the transgenic transplants within 2 weeks.

### Induction of tolerance by bone marrow transplantation

So far, our results indicated that hPLAP-tg rats could serve as a very attractive model for regenerative therapies because of ubiquitous and strong expression of the transgene, and excellent preservation of the marker enzyme in paraffin and MMA sections. However, immune-mediated rejection of hPLAP-tg cells by wild-type rats exposed to cells from transgenic donors made it impossible to use this model for cell tracking. Therefore, we thought of a way to overcome these problems. Previous studies have shown that tolerance against intracellular xenogeneic genes (for example GFP or *neo*, neomycin phosphotransferase) can be induced by busulfan treatment of wild-type C57BL/6 mice, and subsequent transplantation of bone marrow from GFP transgenic C57BL/6 mice [8], or by transplantation of autologous hematopoietic stem cells transduced with a *neo *marker gene in monkeys after total body irradiation [9]. Based on these results, our idea was to combine established methods of tolerance induction to a specific marker with subsequent transplantation of otherwise syngeneic cells labeled with the same marker in order to avoid immune-mediated rejection of the labeled cells.

**Figure 3:**
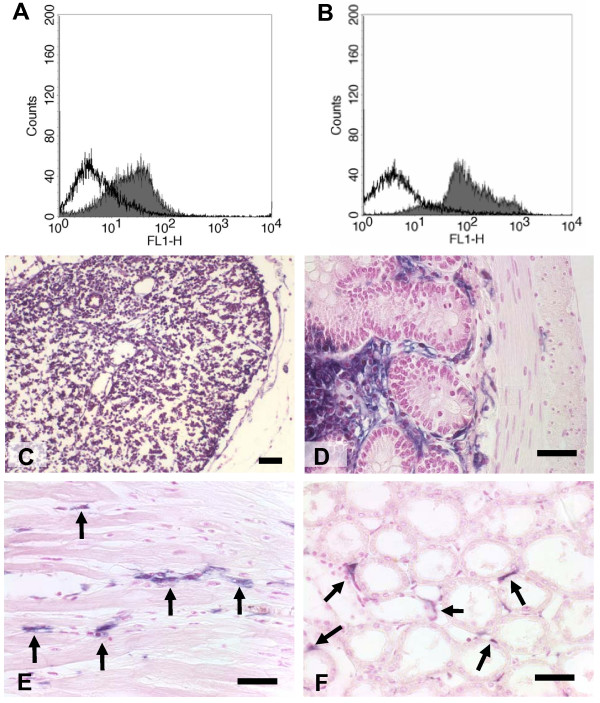
Expression of hPLAP in tissues of irradiated wild-type rats transplanted with bone marrow from hPLAP-tg rats [WT (hPLAP-BMT)], 4 weeks post-transplantation. FACS analysis reveals hPLAP expression (mAb clone 8B6) on peripheral blood leukocytes (A) and bone marrow cells (B) in irradiated WT (hPLAP-BMT) rats (grey area) in contrast to wild-type rats (white area). All leukocytes in lymph nodes demonstrate clear hPLAP staining (C). In cross-sections of the ileum, enterocytes and smooth muscle cells remain unstained, while cells in the submucosa and endothelial cells show intense hPLAP labeling (D). Cardiomyocytes (E) and renal tubular epithelial cells (F) are hPLAP negative cells of recipient origin, while a significant portion of capillary endothelium in heart and kidney is of donor origin as indicated by hPLAP labeling (arrows). Five-μm-thick paraffin sections of abdominal lymph node (C), ileum (D), heart (E), and kidney (F) stained for hPLAP activity and counterstained with nuclear fast red. Bar = 50 μm.

To test the idea of employing marker tolerant animals as a model for regenerative therapies, we initially used lethal irradiation and transplantation of bone marrow from hPLAP-tg F344 rats into wild-type F344 rats as proof-of-principle to prevent the immune response after injection of transgenic cells into wild-type rats. Because the rats are of the same inbred strain, graft-versus-host reactions can be ruled out *a priori*. Fig. 3A–C shows that in irradiated wild-type rats transplanted with hPLAP-tg bone marrow [WT (hPLAP-BMT)] all hematopoietic cells are of donor origin, and stably express hPLAP, 4 weeks post-transplantation. The superb histological detection quality of hPLAP permits a clear distinction between wild-type cells of recipient origin and hPLAP-expressing cells of donor origin (Fig. 3D–F). We found that a significant portion of capillary endothelial cells, but not epithelial or mesenchymal cells such as muscle cells, were of donor origin in heart, kidney, and lung of irradiated WT (hPLAP-BMT) rats (Fig. 3D–F and data not shown).

### Utility of marker tolerant rats for cell tracking in regenerative medicine

**Figure 4:**
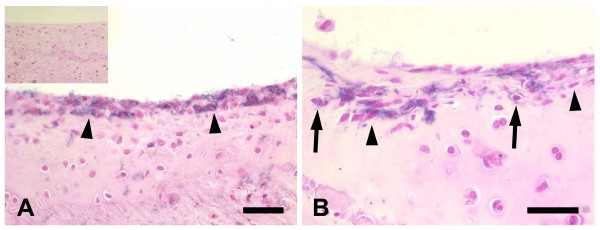
Utility of irradiated, bone marrow-transplanted, marker tolerant rats for regenerative studies and cell tracking. When hPLAP-tg BMC were injected into the knee joint of irradiated WT (hPLAP-BMT) rats, there is a 2 – 3 cells wide layer of donor cells (arrowheads) on the cartilage surface, 1 week post-injection (A). Inset in (A) shows lack of hPLAP-staining in the articular cartilage of an irradiated WT (hPLAP-BMT) control rat not injected with transgenic BMC. Four weeks post-injection, transgenic donor cells have adopted a fibroblast-like (arrowheads) or chondrocyte-like (arrows) appearance, and show incorporation into the surrounding chondrocyte matrix of host origin (B). Five-μm-thick undecalcified MMA sections of ethanol-fixed knee joints stained for hPLAP activity after heat pretreatment and counterstained with nuclear fast red. Bar = 50 μm.

An example of the usefulness of marker tolerant animals for cell therapeutic studies is shown in Fig. 4A and 4B. In this experiment, BMC from hPLAP-tg rats were injected into the knee joint of irradiated WT (hPLAP-BMT) rats. One week post-injection, the transgenic BMC had formed a dense, 2 – 3 cells wide, layer on the intact articular cartilage surface. This finding suggests that these cells underwent proliferation on the cartilage surface earlier. After 4 weeks, the genetically labeled cells displayed a more differentiated fibroblast- or chondrocyte-like phenotype and appeared to have become integrated into the surrounding cartilage tissue of wild-type origin. This experiment underscores the potential usefulness and strengths of this technology, and clearly demonstrates that mesenchymal precursors present in native bone marrow can adhere to the articular cartilage surface and can, subsequently, differentiate into a fibroblastic or chondrocytic phenotype. Although potentially marred by immune-mediated rejection, similar findings have been reported in a goat model of osteoarthritis, in which injection of autologous mesenchymal stem cells harvested from bone marrow, expanded in culture, and transduced with GFP stimulated regeneration of meniscal tissue and retarded progressive joint destruction [11].

**Figure 5:**
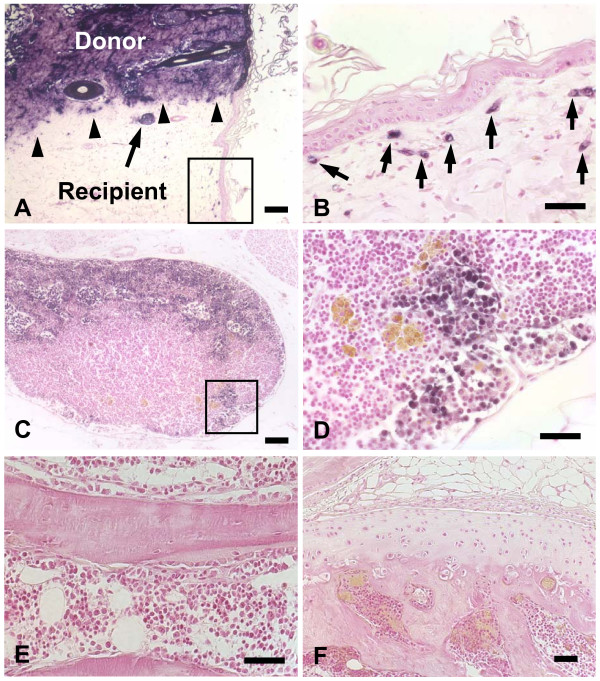
Utility of neonatally tolerized rats for cell tracking. To test whether neonatal exposure of wild-type rats to transgenic cells results in long-lasting tolerance to the marker gene, skin from hPLAP-tg donor was grafted into a neonatally tolerized, 4-month-old F344 wild-type rat. Four weeks post-transplantation, the skin graft from the transgenic donor (upper part of the section) shows strong expression of hPLAP in all cells (A). The border between transgenic donor tissue and wild-type recipient tissue is sharply delineated (arrowheads). A sebaceous gland (arrow in A), and a few capillaries (arrows in B) of transgenic donor origin are found in the surrounding wild-type skin. Keratinocytes do not cross the sharp border between wild-type and transgenic epidermis. Fig. 5B is a high power view of the frame shown in (A). When hPLAP-tg BMC were intravenously injected in neonatally tolerized F344 WT recipients, transgenic donor leukocytes are clearly present in an abdominal lymph node, 2 months post-injection (C-D). Fig. 5D is a high power view of the frame shown in (C). However, hPLAP-tg BMC injected intravenously in neonatally tolerized F344 WT recipients do not home to bone, bone marrow, or cartilage (E-F). Five-μm-thick paraffin or MMA sections of ethanol-fixed skin biopsy (A-B), abdominal lymph node (C-D), tibia (E), and knee joint (F) stained for hPLAP activity after heat pretreatment and counterstained with nuclear fast red. Bar = 100 μm (A, C) or 50 μm (B, D-F).

It is clear that the background of labeled hematopoietic cells is an important shortcoming of irradiated WT (hPLAP-BMT) rats, limiting this model to the study of non-hematopoietic tissues. To test whether neonatal exposure of wild-type F344 rats with cells from hPLAP-tg F344 rats results in a sustainable tolerance to transgenic tissue, we subcutaneously injected neonatal F344 wild-type rats (n = 6) with whole blood from hPLAP-tg rats. Four months later, skin of hPLAP-tg rats was transplanted into these neonatally tolerized rats. The transgenic skin grafts were accepted in 6 out of 6 rats, while a control littermate not having received transgenic blood promptly rejected the transgenic graft. Fig. 5A shows the margin of the transgenic skin transplant in a marker tolerant rat, 4 weeks post-transplantation. It is evident that the border between wild-type and transgenic tissue is sharply delineated, and that capillaries from transgenic donor tissue invade the surrounding wild-type skin (Fig. 5B).

These data clearly demonstrate that sustainable marker tolerance also can be achieved by neonatal exposure of wild-type F344 rats to hPLAP-expressing cells of the same rat strain. Thus, any kind of hPLAP-labeled cell or tissue from F344 rats can be transplanted and studied in these marker tolerant F344 rats in the absence of immune-mediated rejection, and also in the absence of background labeling. To examine the utility of this model for long-term cell tracking, we intravenously injected neonatally tolerized F344 rats with transgenic BMC (n = 4), and assessed the occurrence of hPLAP-labeled cells 2 months after cell administration in various organs. Two months post-injection, we found hPLAP-labeled cells in lymph nodes, but not in liver, heart, kidney, bone, or cartilage (Fig. 5C–F and data not shown), with the exception of extremely rare hPLAP-labeled endothelial cells in heart and kidney (data not shown). hPLAP-stained cells were totally absent in neonatally tolerized F344 controls which had not received transgenic BMC as evidenced by histological and FACS analysis, ruling out the possibility of a background of hPLAP-labeled cells in these rats (data not shown).

## Discussion

In this study we have shown 1) that hPLAP is an excellent marker for histological tracking of genetically labeled cells, 2) that a significant portion of endothelial cells is bone marrow-derived in adult organs, and 3) that by induction of selective tolerance to the marker protein it is possible to track cells in immunocompetent, inbred rats in the total absence of immune-mediated rejection of labeled cells.

In line with previous reports our findings corroborate the notion that hPLAP is a highly suitable marker enzyme for histological tracking of genetically labeled cells in hard and soft tissues [2,4], and we believe that the excellent detection quality of hPLAP will set new standards in histological cell tracking. Using this marker, we showed that a significant portion of capillary endothelial cells were of donor origin in heart, kidney, and lung of WT (hPLAP-BMT) rats, 1 month post-transplantation. There is conflicting evidence whether endothelial cells are of hematopoietic origin in postnatal organisms. Some studies with genetically engineered mice revealed no evidence for a hematopoietic origin of endothelial cells [12]. On the other hand, in mice transplanted with bone marrow from mice homozygous for the genomic globin/pBR322 marker, it has been reported that 0.2 – 1.4% of endothelial cells are bone marrow-derived without tissue injury during 4 months post-transplantation [13]. The much higher portion of labeled endothelial cells found in our study may be due to the higher sensitivity of hPLAP histochemistry compared with the *in situ *hybridization technique necessary for visualization of the globin/pBR322 genomic marker, or due to species differences. Nevertheless, our data strongly suggest that bone marrow is a major source of endothelial precursor cells, in line with the original description of bone marrow-derived endothelial precursor cells and several other reports [14-16]. The reasons why labeled endothelial cells were only very rarely observed 2 months after intravenous BMC injection into neonatally tolerized rats may be 1) the presumably low number of endothelial precursors present in BMC preparations, and 2) a high turnover of endothelial cells in many organs. Labeled endothelial cells could have been replaced by unlabeled bone marrow-derived host cells within the 2-month observation period. In line with the latter hypothesis, labeled endothelial cells can be found at higher frequency in solid organs of neonatally tolerized rats injected with hPLAP-tg BMC, 1 week post-injection (R.G. Erben, unpublished results).

hPLAP-tg rats express hPLAP mainly in the cell membrane of most cell types as demonstrated by FACS analysis in our study. We found that membrane expression of the genetic marker induces strong humoral and cellular immune responses in wild-type recipients that result in the rejection of intravenously infused cells from transgenic donors. It is known from the work of other groups that membrane expression of non-self proteins can elicit immune-mediated rejection involving both B- and T-cell responses in the recipients [6,7]. However, it has become increasingly clear during recent years that immunization phenomena against foreign proteins are not restricted to membrane expression of these proteins. Intracellular expression of foreign proteins also can induce strong T cell-mediated immune responses [8-10]. Therefore, immune-mediated rejection of genetically altered cells is a general problem in transplantation studies using cells labeled with any marker gene, and also in gene therapy protocols. This shortcoming precludes unequivocal interpretation of results from, especially long-term, transplantation experiments with labeled cells.

Here we show that the problem of immune-mediated rejection can be overcome by inducing tolerance to the marker gene by transplantation of hPLAP-tg F344 bone marrow into wild-type F344 recipients, or by exposure of neonatal wild-type F344 rats to blood cells from hPLAP-tg F344 rats. Our experiments have demonstrated that after induction of marker tolerance, transgenic cells and tissues could be successfully engrafted and traced in wild-type rats. As an example of the utility of this concept, we demonstrated hPLAP-labeled cells in lymph nodes, two months after intravenous injection of hPLAP-tg BMC into neonatally tolerized wild-type rats. Thus, we have established a model in which immunocompetent, inbred animals are first made immunologically tolerant to the marker gene that allows tracing of the transplanted cells. In a second step, regenerative therapies with cells labeled with the same marker gene can be tested in the complete absence of immune-mediated rejection in immunocompetent hosts, avoiding the limitations of experiments with immunodeficient SCID (severe combined immunodeficiency) mice or immunosuppressed animals. In immune-privileged compartments such as the central nervous system or in joints, immune-mediated rejection of labeled cells may be delayed. For example, it was reported that mesenchymal cells transduced with GFP survive for up to 12 weeks in the knee joint of goats [11]. Therefore, our finding that hPLAP-tg BMC could be found 4 weeks post-injection in the knee of irradiated WT (hPLAP-BMT) rats does necessarily show that marker tolerant rats are superior to conventional models using wild-type animals for regenerative studies in the knee. Nevertheless, immune-mediated rejection of marker-expressing cells is a potential confounding factor also in immune-privileged compartments in long-term studies.

We used bone marrow transplantation and neonatal induction of tolerance to the marker gene, but a plethora of other strategies for tolerance induction is conceivable. For example, inducible or tissue-specific promoters could be used to reach this aim. Although our experiments were performed using hPLAP as marker gene, the idea to use marker tolerant animals for tracking of labeled cells is applicable to all immunogenic marker proteins.

## Conclusion

Our model is almost fully equivalent to the situation in human patients using autologous adult stem cells for cell and gene therapy, and we believe that hPLAP tolerant inbred or cloned animals will not only be very useful for the development of regenerative therapeutic protocols in the future, but also for all experiments requiring long-term histological tracking of labeled cells such as cell lineage or stem cell experiments in various tissues. In addition, long-term tracking of transplanted cells is a sine qua non to assess the long-term safety of regenerative therapies. Therefore, another strength of this model is its ability to address hitherto unresolved long-term safety issues of cell and gene therapies in immunocompetent animals.

## Methods

### Animals

All experimental procedures were conducted in compliance with prevailing animal welfare regulations. Heterozygous male or female hPLAP-tg F344 rats were mated with wild-type F344 rats, and the resulting wild-type and heterozygous transgenic offspring were genotyped by enzyme histochemistry using a drop of tail blood as described [4]. The rats were housed in pairs at 24°C and a 12 h/12 h light/dark cycle with free access to tap water and commercial rat diets (Altromin 1324 for maintenance and 1314 for breeding, Altromin, Lage, Germany).

### Histology and hPLAP detection

Because hPLAP is sensitive to fixation with formalin [2], tissues and bones were harvested and were fixed in 40% ethanol at 4°C for 48 h. Subsequently, the tissue specimens were dehydrated and embedded in paraffin or in a modified MMA embedding mixture that preserves enzyme activities and can also be used for immunohistochemistry [3]. Five-μm-thick paraffin and MMA sections were cut with a HM360 microtome (Microm, Walldorf, Germany), and were mounted on slides pre-treated with 3-aminopropyltriethoxy-silane (APES, Sigma-Aldrich, Deisenhofen, Germany).

Paraffin sections were deparaffinated using xylene, whereas MMA sections were deplasticized using 2-methoxyethylacetate as described [3]. Deparaffinated and deplasticized sections were rehydrated and heated at 65°C for 30 min in deionized water to block endogenous alkaline phosphatase activity. Sections were then incubated in TRIS buffer (0.1 M Tris-HCl, pH 9.5, 0.1 M NaCl, 5 mM MgCl_2_) containing 0.17 mg/ml of the substrate 5-bromo-4-chloro-3-indolyl phosphate (BCIP, Sigma) at room temperature overnight. Subsequently, sections were counterstained with nuclear fast red (Sigma), dehydrated, and mounted using Vectamount (Vector, Burlingame, CA, USA).

### Flow cytometry

Peripheral whole blood was taken from a tail vein. BMC were harvested from the tibia or femur by short centrifugation of the bone, and were dispersed in PBS by repeated pipetting. For labeling of cell surface hPLAP on peripheral blood and BMC, cell suspensions (approximately 10^6 ^cells each) were incubated with a monoclonal mouse anti-hPLAP antibody (supernatant of clone 8B6, Dako) diluted 1:20 for 30 min on ice. Non-immune mouse IgG_1 _(MOPC-21, Sigma) and cell suspensions from wild-type rats were used as negative controls. After washing twice, the cells were incubated with rat-adsorbed, FITC-labeled goat anti-mouse IgG antibody (Sigma) for 30 min on ice. Prior to FACS analysis, erythrocytes were hemolyzed in blood samples using FACS lysing solution (Becton Dickinson, Heidelberg, Germany). The analyses were performed on a FACScan flow cytometer using CellQuest Pro software (Becton Dickinson, Heidelberg, Germany).

To examine the presence of circulating anti-hPLAP antibodies in serum of wild-type rats that had previously received cells from hPLAP-tg rats, wild-type and transgenic peripheral blood cells were incubated for 30 min on ice with 10 μl undiluted serum from naïve wild-type rats or from wild-type rats that had received transgenic PBL earlier. Using FITC-labeled goat anti-rat IgG antibody (Serotec, Harwell, UK) as secondary antibody, the samples were processed and analyzed as described above.

### Transplantation of transgenic blood and bone marrow cells into wild-type recipients

In order to determine the fate of genetically labeled donor cells in wild-type recipients, we intravenously injected 36 wild-type F344 rats with PBL or BMC (n = 18 each) isolated from hPLAP-tg F344 rats. Transgenic rats were killed by exsanguination from the abdominal aorta under ketamine/xylazine anesthesia. PBL were harvested using a density gradient centrifugation kit (NycoPrep 1.077A, Axis-Shield, Oslo, Norway), washed several times and resuspended in PBS. The percentage of viable cells after the isolation procedure was > 90% (trypan blue exclusion). At the same time, BMC were harvested and washed as described above. After isolation, all cells were resuspended in PBS, and were kept on ice until use. Isolated genetically labeled PBL or BMC were injected intravenously at a dose of 25 × 10^6 ^or 5 × 10^6 ^cells per animal, respectively, into sex-matched wild-type recipients via a lateral tail vein. All recipients received daily injections of genetically labeled cells over 7 consecutive days (days 1 – 7). The recipient rats were killed on days 8, 15, 22, or 28 (n = 4 – 6 each for PBL and BMC recipients) by exsanguination from the abdominal aorta under ketamine/xylazine anesthesia. From all recipient animals a variety of soft tissues and bones were harvested, fixed in 40% ethanol at 4°C for 48 hours, and processed as described above.

### Humoral and cellular immune response to transgenic cells

To examine the development of antibody production against transgenic cells, we repeatedly exposed wild-type rats to PBL from hPLAP-tg donors. The PBL were harvested from hPLAP-tg rats as described above, and resuspended in PBS. Subsequently, 1 × 10^6 ^PBL were subcutaneously or intraperitoneally injected three times into wild-type F344 rats (1 rat each) with an 11-day interval between the injections. At baseline, and 10 days after each injection, blood was drawn from a tail vein of the wild-type recipients to obtain serum. For mixed lymphocyte reaction, spleen cells were isolated by density gradient centrifugation (NycoPrep 1.077A). The spleen cells from naïve wild-type rats or from wild-type rats previously exposed to transgenic cells (3 intraperitoneal injections at 10-day intervals) were co-cultured with transgenic spleen cells for 5 days at a total density of 5 × 10^5 ^cells per well (2.5 × 10^5 ^wild-type and transgenic cells each). During the last 18 hours of culture, ^3^H thymidine (2 Ci/mmol) was added to quantify cell proliferation. ConA (10 μg/ml) was used as a positive control.

### Lethal irradiation and bone marrow transplantation

To transplant bone marrow from hPLAP-tg rats into wild-type F344 rats, wild-type animals (n = 6) were lethally irradiated with a dosage of 8.5 Gy at 0.9473 Gy/min using a cobalt-60 irradiator (Eldorado, Atomic Energy of Canada, Ottawa, Canada). Four hours after the irradiation, the rats were intravenously injected with 3 – 4 million BMC isolated from hPLAP-tg rats as described above. To rule out unsuccessful engraftments, the same injection of freshly prepared transgenic BMC was repeated 24 hours after the irradiation.

### Intraarticular injection of genetically labeled cells

Next, we wanted to explore whether intraarticularly injected BMC from hPLAP-tg rats would survive, proliferate, and differentiate in the knee joint of irradiated WT (hPLAP-BMT) rats. For this experiment 6 male and female wild-type F344 rats at the age of 3 months were lethally irradiated and transplanted with BMC from hPLAP-tg F344 rats. Two weeks after the bone marrow transplantation, 5 × 10^6 ^BMC isolated from hPLAP-tg rats were injected once into the cavity of the left or right knee joint under medetomidine/midazolam/fentanyl anesthesia. The rats were killed 7 days or 28 days (n = 3 each) after the intraarticular injection by exsanguination from the abdominal aorta under ketamine/xylazine anesthesia. The intact knee joints were harvested, trimmed, fixed in 40% ethanol at 4°C for 48 hours, and embedded in MMA. Frontal 5-μm-thick sections were cut as described above.

### Induction of tolerance by neonatal exposure to transgenic cells

To induce tolerance to the marker gene by an alternative method, 6 neonatal wild-type F344 rats pups, within 4 hours after birth, were subcutaneously injected once with 20 μl of whole blood from hPLAP-tg F344 donors. One control litter mate did not receive transgenic cells. Four months later, all 7 rats received skin grafts (approx. 1 cm^2^) from sex-matched hPLAP-tg donors at the back under anesthesia with medetomidine/midazolam/fentanyl. The grafts were controlled daily. Four weeks post-transplantation, biopsies were taken under anesthesia from the margin of the skin grafts, fixed in 40% ethanol at 4°C for 48 hours, embedded in paraffin, sectioned at 5 μm thickness, and stained for hPLAP enzyme activity as described above.

### Tracking of transgenic cells in neonatally tolerized rats

Tolerance to hPLAP in F344 WT hosts was induced and confirmed by skin grafts as described above. At the age of 4 months, 4 neonatally tolerized WT rats were intravenously injected once with 10^8 ^BMC from hPLAP-tg donors, and were killed 2 months post-injection. Various tissues were harvested, fixed in 40% ethanol at 4°C for 48 hours, embedded in paraffin or MMA, sectioned at 5 μm thickness, and stained for hPLAP enzyme activity as described above. Neonatally tolerized control rats were killed without prior injection of labeled cells to rule out a background of labeled cells in the marker tolerant recipients.

### Statistical analysis

Statistics were computed using SPSS for Windows 14.0 (SPSS, Chicago, IL). The data of the mixed lymphocytes reaction experiments were analyzed using one-way analysis of variance (ANOVA). When the analysis of variance performed over all groups indicated a significant (p < 0.05) difference among the groups, statistical differences between two groups were subsequently evaluated with Fisher's least significant difference test. P values of less than 0.05 were considered significant. The data in Figure 2D are given as means ± SEM.

## Authors' contributions

KIO and RGE designed research. KIO, NJU, and KW performed research. KIO, NJU, KW, and RGE analyzed data. EPS provided the rats for these experiments. KIO, NJU, and RGE wrote the manuscript. All authors read and approved the final manuscript.
